# Rhodopsin Molecular Evolution in Mammals Inhabiting Low Light Environments

**DOI:** 10.1371/journal.pone.0008326

**Published:** 2009-12-16

**Authors:** Huabin Zhao, Binghua Ru, Emma C. Teeling, Christopher G. Faulkes, Shuyi Zhang, Stephen J. Rossiter

**Affiliations:** 1 School of Life Sciences, East China Normal University, Shanghai, China; 2 UCD School of Biology and Environmental Science and UCD Conway Institute of Biomolecular and Biomedical Research, University College Dublin, Belfield, Dublin, Ireland; 3 School of Biological and Chemical Sciences, Queen Mary University of London, London, United Kingdom; University of Stellenbosch, South Africa

## Abstract

The ecological radiation of mammals to inhabit a variety of light environments is largely attributed to adaptive changes in their visual systems. Visual capabilities are conferred by anatomical features of the eyes as well as the combination and properties of their constituent light sensitive pigments. To test whether evolutionary switches to different niches characterized by dim-light conditions coincided with molecular adaptation of the rod pigment rhodopsin, we sequenced the rhodopsin gene in twenty-two mammals including several bats and subterranean mole-rats. We compared these to thirty-seven published mammal rhodopsin sequences, from species with divergent visual ecologies, including nocturnal, diurnal and aquatic groups. All taxa possessed an intact functional rhodopsin; however, phylogenetic tree reconstruction recovered a gene tree in which rodents were not monophyletic, and also in which echolocating bats formed a monophyletic group. These conflicts with the species tree appear to stem from accelerated evolution in these groups, both of which inhabit low light environments. Selection tests confirmed divergent selection pressures in the clades of subterranean rodents and bats, as well as in marine mammals that live in turbid conditions. We also found evidence of divergent selection pressures among groups of bats with different sensory modalities based on vision and echolocation. Sliding window analyses suggest most changes occur in transmembrane domains, particularly obvious within the pinnipeds; however, we found no obvious pattern between photopic niche and predicted spectral sensitivity based on known critical amino acids. This study indicates that the independent evolution of rhodopsin vision in ecologically specialised groups of mammals has involved molecular evolution at the sequence level, though such changes might not mediate spectral sensitivity directly.

## Introduction

Mammals are arguably the most ecologically diverse group of vertebrates, having radiated to fill a diverse range of niches from the deep ocean to the night sky. Such diversification has involved considerable adaptive changes in their sensory systems. Mammal species are typically highly visual, with vision playing important roles in sexual selection, foraging behaviour and predator avoidance [1]–[5]. It is thus perhaps unsurprising that the visual systems of mammals show numerous adaptations for inhabiting different light conditions. Such specializations include the anatomical divergence of the lens, iris, pupil and cornea [6], [7], the presence of a reflective layer, and the distribution, combination and properties of the constituent light sensitive pigments [8], [9].

Light sensitive pigments comprise a membrane bound G-protein-coupled receptor (GPCR) known as an opsin and a chromophore group (typically 11-*cis* retinal in mammals) [10]. Absorption of light results in photoisomerisation of the chromophore, which induces conformation changes in the opsin that leads to signal transduction. Most mammals possess three classes of opsins, which differ in their absorption spectra. The SWS1 (short-wavelength sensitive type 1) and M/LWS (middle/long-wavelength sensitive) are restricted to cone photoreceptor cells and are typically responsible for color vision in bright light [8], [11], whereas rhodopsin occurs on the rod cells and is extremely sensitive, so enabling dim light (scotopic) vision [8], [9].

Reconstruction of opsin and rhodopsin proteins have shown that their absorption spectra are determined by a number of key amino acid residues, and that these sites occur in the protein's seven transmembrane (TM) α helices [8], [12]–[13]. Subsequent critical site replacements have usually been explained in the context of evolutionary adaptations to different light environments [14]–[17]. For example, the independent evolution of ultra-violet sensitivity in the opsins of some birds, amphibians and mammals, might have arisen to improve visual contrast detection and avoid UV damage, while the regain of UV vision in some birds has been linked to migration behaviour triggered by day length [18]. Moreover, a number of mammalian lineages (e.g. horseshoe bats, cetaceans) have completely lost one of their opsin genes, which appears to result from a relaxation in selection [19]–[22].

Relatively fewer studies have been undertaken on the molecular evolution of vertebrate rhodopsin genes in spite of its key function in conferring monochromatic vision in low light. The molecular mechanism of spectral tuning in rhodopsin appears to be influenced by 13 amino acids [17], [23]–[24]. Shifts in spectral tuning appear to correlate with foraging depth in marine mammals [14] and have also been linked to differential light environments in some fishes [15], [25]–[28]. Darwinian selection along the rhodopsin gene has been detected during the adaptive radiation of cichlid fishes [15]. In this study we present the most detailed comparative phylogenetic study of mammal rhodopsins to date. We include several groups that are highly specialized for living in low light conditions, including bats, subterranean mole-rats, pinnipeds and cetaceans. We test the hypotheses that the rhodopsin gene has undergone molecular adaptation associated with evolutionary switches to different niches characterized by low light conditions, and, more specifically, that these changes will have coincided with losses of the *SWS1* gene. In addition, we undertake a more detailed study of rhodopsin evolution among several clades of bats that use different sensory modalities and in which *SWS1* has undergone differential psuedogenisation among lineages [22].

## Results

### New Rhodopsin Gene Sequences

We sequenced approximately 3.3 kb of the rhodopsin gene from 22 mammal species and analysed our new data along with the published sequences of an additional 37 mammal species. All new sequences were found to have strictly conserved intronic-splice signals (GT/AG) and, based on the amplification of mRNA from seven bat species, we found no differences between genomic DNA and coding sequences. In total, we obtained 983 bp of genomic DNA for comparative analyses, representing 94% of the coding sequence (1047 bp) and including all transmembrane (TM) helical regions, as well as extracellular domains implicated in the function of visual pigments [29]. We identified 327 amino acids and no premature stop codons were detected. With one exception, none of the new sequences contained insertions or deletions when compared to the 37 published sequences on GenBank. However, the afrotherian Hottentot golden mole showed one 3 basepair deletion that was in frame.

An alignment of 327 amino acids showed that only 52 sites (∼15.9%) were variable and most functionally important residues were highly conserved (Figure S1, Supplementary Material online). These conserved sites included the Schiff base formation of K296 [30], the E113 residue of the Schiff base counterion [31], the disulfide linkage of C110 and C187 [32], and three sites that are implicated in palmitoylation (C140, C322 and C323) [33]. The positions of these amino acid positions here and throughout the paper are numbered according to the bovine rhodopsin [29].

### Phylogenetic Reconstruction

**Figure 1:**
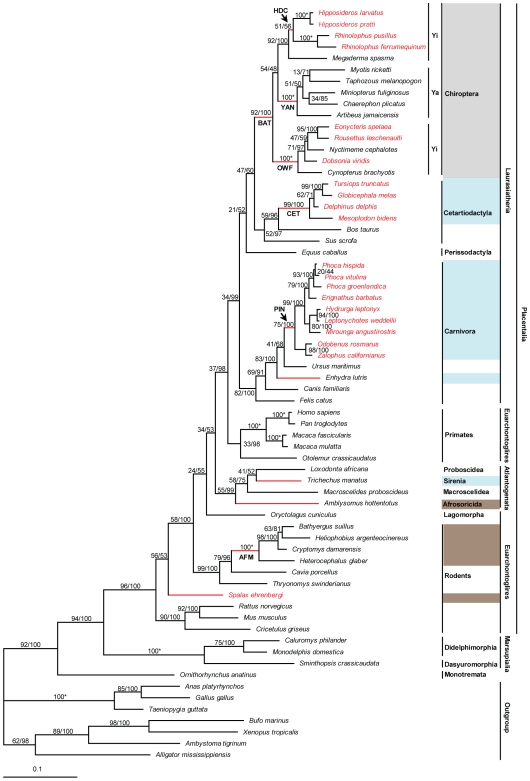
Putative gene tree for rhodopsin using ML and Bayesian approaches with no constraints on the topology. Branch lengths are scaled by the number of nucleotide substitutions per site. Numbers at the nodes are ML bootstrap values/Bayesian posterior probabilities. 100* represents both 100% ML bootstrap support and a posterior probability of 1. Focal branches examined in our selection tests are shown in red. These comprise the ancestral branches to the high-duty-cycle echolocating bats (HDC), the Yangochiroptera (YAN), the Chiroptera (BAT), the Old World fruit bats (OWF), the cetaceans (CET), the pinnipeds (PIN) the African mole-rats (AFM) as well as to ancestral branches to four individual taxa (sea otter, West Indian manatee, Hottentot golden mole and Middle East blind mole-rat). Within the bats, Yinpterochiroptera is coded as Yi and Yangochiroptera as Ya. In addition, the names of taxa known to have lost their SWS1 opsin are shown in red, and their corresponding photopic niches are colour coded as brown for subterranean and light blue for aquatic. Finally, all bats have been shaded grey.

We combined the new rhodopsin sequences with published data and undertook phylogenetic reconstruction of rhodopsin gene sequences for 59 mammals, including groups that have evolved to occupy subterranean, aquatic and nocturnal niches (summarised in Figure 1). The unconstrained phylogenetic tree with ML bootstrap values and Bayesian posterior probabilities is shown in Figure 1. Although some major clades were strongly supported (bats and Placentalia), the overall rhodopsin phylogeny was not completely consistent with the published species tree [34], [35]. The main deviations from the species topology were seen in the rodents, in which members of the Hystricomorpha (African mole-rats and allies) were now basal to the other placentals (including Myomorpha), and in the Yinpterochiroptera, in which fruit bats (Pteropodidae) were now basal to the echolocating taxa (horseshoe bats and allies + Yangochiroptera). This putative gene tree topology was also recovered when the phylogenetic analyses were repeated using the same data but excluding the 13 critical amino acids (data not shown), indicating that support for this phylogenetic signal is contained elsewhere in the gene.

Our Shimodaria-Hasgawa tests revealed significant differences between the unconstrained putative gene tree and the species tree (Table 1). However, when we forced either Rodentia monophyly or Yinpterochiroptera monophyly in the gene tree, there was no significant difference from the species tree (Table 1). This result suggests that statistical differences between our gene tree and the true species tree can be traced to these groups, which appear to have experienced accelerated evolution.

**Table 1:** Species versus gene tree for the mammalian rhodopsin data (*P<0.05).

Topology Tests	Log likelihood scores	Δ in –ln likelihood	*P* values for SH tests
Species tree	14231.37		
Species tree versus Gene tree	14298.79	67.42	0.001*
Species tree versus Gene tree + Rodentia constrained	14240.97	9.60	0.493
Species tree versus Gene tree + Yinpterochiroptera constrained	14243.09	11.72	0.420

### Spectral Tuning of Extant and Ancestral Taxa

We examined the following 13 amino acid sites that have been linked to spectral tuning in rhodopsin: 83, 96, 102, 122, 183, 194, 195, 253, 261, 289, 292, 299 and 317 [17], [23]–[24] (listed in Table S4). We inferred the wavelength of maximum absorption (λ_max_) conferred by particular combinations of critical amino acids following published studies [14], [17], [23]–[24] (see Table S4). Specifically, we assumed that the single amino acid replacements D83N, M183L, S299A would lead to downward shift of λ_max_ by 2 nm based on data from pinnipeds [24], and we assumed that the single mutation L194P and double mutations D83N/L194P would result in downward shift of 3 and 5 nm, respectively, based on cetaceans [14].

Therefore, the λ_max_ values of the three bats *Rhinolophus pusillus*, *R. ferrumequinum* and *Miniopterus fuliginosus* were inferred to be 499 nm, whereas that of *Myotis ricketti* was inferred as 497 nm with the 13 critical sites identical to those of the African elephant [36]. Other bats did not differ from the mammalian consensus compliment of critical sites, and were assumed to have a λ_max_ of 501 nm (Table S4 and Figure S2, Supplementary Material online).

Of the African mole-rats (Family: Bathyergidae), two taxa (*Bathyergus suillus* and *Heterocephalus glaber*) shared the substitution L194P and had an inferred λ_max_ of 498 nm, whereas the other two (*Cryptomys damarensis* and *Heliophobius argenteocinereus*) shared the double mutations D83N and L194P, and so their λ_max_ was estimated to be 496 nm. For two species (the Hottentot golden mole and the horse), data on two critical sites were not available; however, these sites were conserved across all other mammals, and so we assumed that their λ_max_ was 501 and 499 nm, respectively (Table S4, Figure S2, Supplementary Material online).

Reconstructed ancestral rhodopsins at each node based on Maximum Likelihood and Maximum Parsimony approaches gave similar results, with most nodes having the 13 key amino acids of the mammalian consensus sequence (see Table S4 and Figure S2, Supplementary Material online). Consequently, these ancestral proteins were inferred to have a λ_max_ of 501 nm. However, some replacements occurred in some species of pinniped, cetacean, bat and African mole-rat, with associated downward shifts ranging from 2 to 17 nm (Figure S2, Supplementary Material online).

### Tests for Selection

We applied codon based models to test for heterogeneous selection pressures acting on the rhodopsin gene across the mammal phylogenetic tree. We focused on several groups that inhabit low photopic environments, including cetaceans, pinnipeds, bats and molerats, some of which have lost their SWS1 opsins (summarized in Figure 1). In addition, we undertook a more focused study on selection among clades of bats that exhibit contrasting sensory modalities based on vision and echolocation [22]. The results of model comparisons with likelihood ratio tests are given in Table 2 and full details of all model parameters are given in Table S3.

**Table 2:** Likelihood ratio tests (LRTs) for selection tests.

Comparisons	2*Δℓ*	df	*P*-value
Dataset I: all mammals			
*Branch Models*			
A1: One-ratio vs. B1: Free-ratio	329.601	115	**<0.001**
A1: One-ratio vs. C1: Two-ratio (AFM branch, background)	3.835	1	0.050
A1: One-ratio vs. D1: Two-ratio (Middle East blind mole-rat branch, background)	0.559	1	0.46
A1: One-ratio vs. E1: Two-ratio (Hottentot golden mole branch, background)	0	1	1
A1: One-ratio vs. F1: Two-ratio (CET branch, background)	8.368	1	**0.004**
A1: One-ratio vs. G1: Two-ratio (PIN branch, background)	0.663	1	0.416
A1: One-ratio vs. H1: Two-ratio (West Indian manatee branch, background)	5.738	1	**0.017**
A1: One-ratio vs. I1: Two-ratio (sea otter branch, background)	0.113	1	0.736
A1: One-ratio vs. J1: Two-ratio (BAT branch, background)	0	1	1
*Site Models*			
K1: M1a: nearly neutral vs. L1: M2a: positive selection	0	2	1
M1: M8a: β & ω = 1 vs. N1: M8: β & ω	0	2	1
*Clade Models*			
A1: M1a vs. O1: Model C (all African mole-rats)	363.146	3	**<0.001**
A1: M1a vs. P1: Model C (all pinnipeds)	398.453	3	**<0.001**
A1: M1a vs. Q1: Model C (all cetaceans)	393.407	3	**<0.001**
A1: M1a vs. R1: Model C (all bats)	378.865	3	**<0.001**
Dataset II: Bats only			
*Branch Models*			
A2: One-ratio vs. B2: Free-ratio	35.9	26	0.094
A2: One-ratio vs. C2: Two-ratio: OWF (Old World fruit bats) branch, background	0.52	1	0.471
A2: One-ratio vs. D2: Two-ratio: HDC (high-duty-cycle bats) branch, background	2.34	1	0.126
A2: One-ratio vs. E2: Two-ratio: YAN (Yangochiroptera) branch, background	4.58	1	**0.032**
*Site Models*			
F2: M1a: nearly neutral vs. G2: M2a: positive selection	0	2	1
H2: M8a: β & ω = 1 vs. I2: M8: β & ω	0	2	1
*Clade Models*			
F2: M1a: nearly neutral vs. J2: Model C: all Old World fruit bats	11.06	3	**0.011**
F2: M1a: nearly neutral vs. K2: Model C: all high-duty-cycle bats	11.1	3	**0.011**
F2: M1a: nearly neutral vs. L2: Model C: all Yangochiroptera	11.5	3	**0.009**

Twice difference of likelihood values between two nested model is shown as 2*Δℓ*; the degrees of freedom are abbreviated as df; and significant *P*-values (<0.05) are indicated in bold. Codes for focal branches are listed in Figure 1 (e.g. AFM) and the parameters of the models (A1 to R1 and A2 to L2) are given in Table S3).

The estimates of ω (the ratio of the non-synonymous substitution rate to the synonymous substitution rate) based on a one-ratio model was 0.040 for all mammals (see supplementary Table S3), suggesting strong gene conservation across the tree. Moreover, site models (M2a and M8) failed to detect positive selection or identify any individual sites with ω>1 (see supplementary Table S3, Supplementary Material online). In contrast, a free-ratio model for all mammals did fit the data significantly better than the corresponding one-ratio model, suggesting that heterogeneous selective pressures might occur along one or more specific evolutionary lineages. This was confirmed by a series of two-ratio branch models, in which each foreground branch of interest was in turn allowed to have a different ratio from the rest of the tree (background). These models were applied to test several species or ancestral branches that are associated with poor photopic environments, as described below.

In the dataset of all mammals (I), models F1 (cetaceans versus background) and I1 (West Indian manatee versus background) were significantly better fits to the data than the one-ratio model (A1). However, while the ω value estimated for branch CET (ancestral to cetaceans) was around five times higher than the background, that of the West Indian manatee branch was actually lower (0.009 versus 0.041). It is also noteworthy that the foreground ω estimate (0.104) of the branch ancestral to African mole-rats (AFM) was found to be nearly three times higher than the background, and the associated likelihood ratio test was on the margin of statistical significance (*P* = 0.05) (Table 2). Finally, we also tested the lineage of the elephant seal, because it has been previously shown to have spectral tuning to blue wavelengths of light [37], and this was also found to be significant (data not shown).

In the dataset of bats only (II), the ω values for branches OWF (ancestral to Old World fruit bats) and HDC (ancestral to high-duty-cycle echolocators) were not significantly different from the background (Table 2). This result is consistent with the comparison between the free-ratio and one-ratio models, which also showed no significant difference and suggested no heterogeneous selective pressures along bat lineages (Table 2). The ω ratio of the branch ancestral to the Yangochiroptera (YAN) was estimated to be significantly lower than the background (Table 2); however, this difference was not detected when we repeated the same test under the species tree topology (data not shown), and thus this result appears not be robust.

Separate clade models undertaken for African mole-rats, pinnipeds, cetaceans and bats all showed evidence of significant divergent selection. Estimates of ω were higher in the foreground (focal clade) than in the background for African molerats (0.298 versus 0.199, respectively), pinnipeds (1.262 versus 0.197, respectively) and cetacean (1.205 versus 0.180, respectively). In the latter two cases, the ω was greater than one, suggesting positive selection in these clades. However, in the bat clade, the ω ratio was estimated to be lower than the background (0.102 versus 0.236, respectively).

Our analyses of three clades of bats that exhibit contrasting sensory modalities also revealed significantly different selection pressures. We found that members of the Old World fruit bat clade had a ω value similar to other bats (0.267 versus 0.297, respectively), while bats with high-duty-cycle echolocation had a significantly higher ω value than other bats (0.339 versus 0.273, respectively), and bats with low-duty-cycle echolocation had a significantly lower ω value than other bats (0.121 versus 0.245, respectively). In all three model comparisons, 5.6% to 6.5% of sites were identified as being under divergent selection (see details in Table 2 and supplementary Table S3, Supplementary Material online).

We repeated all the selection tests using the species tree topology and obtained similar results as the putative gene tree. The only case that differed was the comparison between the one-ratio model and the two-ratio model in which the Yangochiroptera (YAN) ancestral branch was the foreground. In this case, the LRT became non-significant (results not shown).

### Sliding Window Analyses

**Figure 2:**
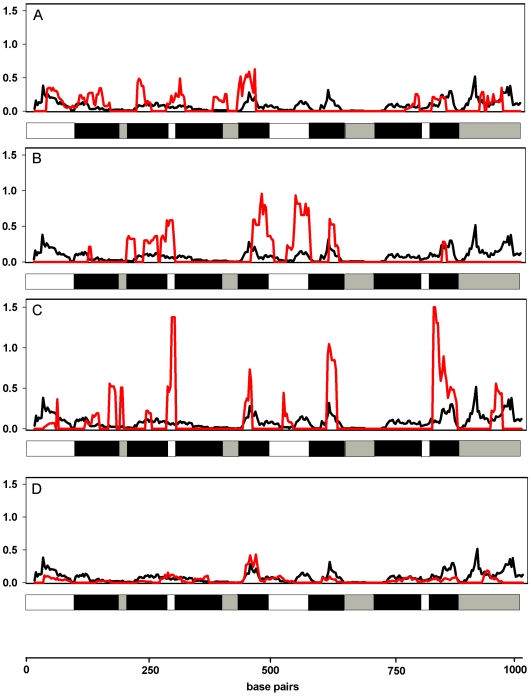
Sliding window analysis (window size  = 30 bp, step size  = 3 bp) to show variation in omega value (dN/dS) along the rhodopsin gene, between all mammals (black) and, in red, (A) African mole-rats, (B) cetaceans, (C) pinnipeds and (D) bats. Beneath each plot is a schematic of the rhodopsin gene, which illustrates the distribution of transmembrane domains (black), extracellular (white) and intracellular (grey) domains.

The results of sliding window analyses are presented in Figure 2. Estimates of ω values were found to be low for the alignment of rhodopsin coding sequences for all mammals (shown in black), suggesting purifying selection as the main force during rhodopsin evolution. However, higher ω estimates were found in African mole-rats, cetaceans and pinnipeds (Figure 2A, B and C), indicative of elevated evolutionary rates. In pinnipeds, the ω ratio exceeded one in two regions, suggesting positive selection. All of the regions with higher ω values were transmembrane and extracellular domains. In bats, ω ratios were not obviously greater than in mammals in general (Figure 2D), though ω ratios were elevated in high-duty-cycle echolocating bats (data not shown).

## Discussion

We undertook phylogenetic and molecular analyses of fifty-nine species of mammal to test whether visual adaptation to low light conditions is associated with molecular adaptation in the rod pigment rhodopsin. Our analyses included the members of several clades that have independently switched to different forms of ecological niche characterized by low light, including cetaceans, pinnipeds, bats and subterranean mole-rats, as well as the West Indian manatee, Hottentot golden mole and sea otter.

All new and published gene sequences were intact with no premature stop codons or frameshift mutations, which, together with the evidence of expression of mRNA in bat retinae, strongly indicate that these mammals have a functional rhodopsin protein. Indeed, analyses of substitution rates indicate that the rhodopsin gene has been predominantly subject to purifying selection during the diversification of mammals, with lower estimates of d_N_/d_S_ (ω) based on one-ratio models than the average (0.173) reported for mammalian nuclear genes [38]. Moreover, site models failed to detect heterogeneous selective pressure among sites. By comparison, several branches and clades did show evidence of accelerated evolution or divergent selection when compared to other groups. In general, more clade models were significant than ancestral branch models, even where these corresponded to the same taxa. This discrepancy is likely to reflect the increased power of the clade tests, which consider multiple lineages and so have a greater chance of detecting informative changes.

In the African mole-rats, the higher estimated ω ratio along the ancestral branch, and the greater ω value for the clade (see supplementary Table S3, Supplementary Material online) suggest that the rhodopsin gene has evolved relatively rapidly in this group. Given the ecology of this group, it is tempting to afford such accelerated evolution to relaxed selection associated with living underground. Indeed, the eyes of African mole-rats are vestigial, and, in some species, the visual subsystems are severely reduced [39]. Moreover, members of this group are known to rely heavily on olfactory and tactile senses for short-distance orientation, and detect seismic signals for long-distance communication [40]–[42], indicating that vision might not be essential. However, in spite of these points, it is important to note that African mole-rats have been found to possess more cones (representing ∼10% of the photoreceptors) than other nocturnal rodents, the inferred adaptation to discriminate bright light has been attributed to circadian rhythm entrainment rather than scotopic vision [39]. A role in photo-entrainment could also explain the retention of the functional gene in the African bathergid mole-rats as well as the Middle East blind mole-rat and the Hottentot golden mole, both of which possess subcutaneous eyes, and have independently evolved to occupy a subterranean niche. Therefore, at this time, we cannot rule out the possibility that the elevated ω ratio in the rhodopsin of mole-rats reflects a past burst of positive selection rather than relaxed selection.

By comparison, elevated ω ratios detected in clades of both cetaceans and pinnipeds (see supplementary Table S3, Supplementary Material online) are more likely to have some adaptive significance in vision. Consistent with living in low light conditions (rather than complete darkness), the retinae of both groups have been found to be highly rod-dominated with only 0.4%–2% of photoreceptors represented by cones [43]. Moreover, behavioural studies of members of these groups suggest they are functionally dichromatic [44]–[46] despite the fact that recent genetic [20], [24] and immuonocytochemical [43] evidence reveals that cetaceans and pinnipid species have typically lost their blue cones (reviewed by [47]. It has been suggested that without SWS1 opsins, these animals discriminate color by comparing the signals from the green cones and rods (see [48]. Consequently, the rhodopsin gene in these clades might have undergone molecular adaptation to confer dichromatic vision in low light. In constrast, the sea otter and West Indian manatee had a similar and lower ω value than other mammals, respectively. Since both species spend considerable time near the water surface and also possess a functional SWS1 opsin, it seems probable that their rhodopsin genes have predominantly been subject to purifying selection, as appears to be the case for most mammals. Indeed, the visual system of the manatee appears to be morphologically similar to terrestrial mammals [7].

In the bats, rhodopsin homologues in species with and without laryngeal echolocation were characterized by similar ω ratios, in spite of the fact that the latter (Old World fruit bats) are characterized by larger eyes and are often considered to be more dependent on low light (scotopic) vision. However, evidence of divergent selection was found between these groups. Interestingly, clade models suggested that bats that have evolved high-duty-cycle echolocation had a significantly higher ω ratio than other bats (i.e. Old World fruit bats and low-duty-cycle echolocators) (see Supplementary Table S3, Supplementary Material online). In the absence of positive selection, one possibility is that high-duty-cycle echolocators have experienced relaxed selection, perhaps due to a relatively higher dependence on the auditory system. Interestingly these results from bat rhodopsin genes show parallels with the recent results of two other studies of sensory genes in bats. Genetic analyses of medium and shortwave opsins showed that species with high-duty-cycle echolocation have also lost their *SWS1* genes via both frame shift and nonsense mutations [22]. Moreover, positive selection on the ancestral branch of this group has also been detected in the *Prestin* gene, which encodes a motor protein implicated in high frequency hearing that is especially characteristic of this group [49]. Such concordance indicates that multiple genes are impacted by common selection pressures, and raises the possibility that molecular changes at one sensory gene will have direct consequences for genes controlling the same or other sensory modalities, perhaps via trade-offs (see [22]).

Despite the divergent selection pressures reported here, we could find no clear evidence that the rhodopsin gene in mammals inhabiting low light conditions had undergone consistent spectral tuning at known critical amino acid sites (Table 2), though these inferences need to be substantiated by mutagenesis of synthesized proteins. Previously, dim-light vision in vertebrates has been classified into deep-sea (479–486 nm), intermediate (491–496 nm), surface (500–507 nm) and red-shifted (∼525 nm), based on the inferred peak sensitivity (λ_max_) of their rhodopsin, as well as considerations of life history and ecology [17]. Accordingly, all bats examined appeared to possess a rhodopsin that ranges in sensitivity from 497 to 501 nm, thus overlapping with some surface fishes. Similarly, rhodopsin in African mole-rats can be classified as either surface or intermediate types (496–498 nm), which are slightly blue-shifted compared to the phylogentically distinct subterranean Middle East blind mole rat and Hottentot golden mole (both 501 nm). Furthermore, we found similar predicted sensitivities for the rhodopsin gene in a range of marsupials (499 and 501 nm), murid rodents (501 nm) and primates (497–501 nm), as well as the elephant shrew (501 nm) and horse (501 nm). The largest shifts in spectral tuning appear to occur in some marine mammals, as previously reported based on electroretinogram measurements [24]. The spectral-tuning properties of cetacean rhodopsins have been linked to foraging depth [14] and, of the four cetaceans studied here, Sowerby's beaked whale has the deep-sea type of rhodopsin (λ_max_ of 484 nm), while the others possess rhodopsins with λ_max_ of 489 nm (Table 2), which are more likely to be classified as the intermediate type. In contrast, most pinnipeds possess a surface rhodopsin with λ_max_s ∼500 nm (Table 2), though the Northern elephant seal has a deep-sea rhodopsin with a λ_max_ of 483 nm.

Yet even without spectral shifts, our sliding window analyses indicate that most amino acid replacements in the mammal rhodopsin gene are concentrated in several key domains, pointing to functional significance. Extracellular domain I comprises just six amino acids and includes the replacement V104I that is seen in the leopard seal, Weddell seal, the high-duty-cycle bats, western long-fingered bat, Sowerby's beaked whale and Cape dune mole rat. However, it is unlikely that this site confers any spectral shift [17]. The transmembrane helix VII spans 21 amino acids and has accumulated numerous non-synonymous substitutions including I286T that was only recorded in the leopard and Weddell seal, S297A only in the harp, harbor and ringed seal, and S297G in the bearded seal. Other replacements (A292S, S298A and S299A) were shared across phylogentically distant several taxa. Of these, A292S is a critical site replacement, S298A and S299A are unlikely to cause spectral-tuning, while the spectral properties of replacements at positions 286 and 297 are not clear [17]. Indeed, transmembrane and extracellular domains often interact with ligands [50] and, in G-protein-coupled receptors in general, appear to bind small molecules [51], [52] and larger ligands [50], [53]–[54]. However, no such interactions between ligands and extracellular domains have been documented in rhodopsin specifically. Consequently, unless these replacements have some unknown adaptive significance for rhodopsin function, such as in phototransduction, then it is not possible to dismiss some degree of neutral variation.

To conclude, our results indicate that rhodopsin has undergone divergent selection pressures in several groups of mammal that inhabit low light conditions, and that cases of accelerated evolution are likely to be adaptive for vision at low light (cetaceans and pinnipeds) and, perhaps, photo-entrainment. In two groups (bats and rodents), variation in selection pressures appear to have contributed to conflicts between the species tree and putative gene tree, highlighting the potential pitfalls of using functional genes to reconstruct phylogenetic histories (see also [49]). More work is now needed to determine whether the amino acid differences observed among mammals with divergent selection signatures do indeed impact on the strength and pattern of receptor-ligand interactions and also whether other critical sites for spectral tuning exist in the Rhodopsin protein.

## Materials and Methods

### Data Collection and Taxon Coverage

We generated new rhodopsin coding sequences (∼3.3 kb) for 22 mammal species and combined these with 37 existing mammalian sequences, providing both wide taxonomic coverage from across the tree and detailed representation of several key groups associated with low light conditions, and, in some cases, the loss of shortwave opsin (taxa listed in Table S1, Supplementary Material online).

For nocturnal taxa, we sequenced 15 species of bat (Order Chiroptera) comprising five non-echolocating fruit bats, four species that exhibit high-duty-cycle echolocation and six that exhibit low-duty-cycle echolocation [55]. These two forms of echolocation are broadly found in separate divergent clades [56]. For subterranean taxa, we sequenced three species of African mole-rat as well as the related non-burrowing cane rat. We also sequenced a subterranean afrotherian (golden mole) and, for comparison, a non-burrowing afrotherian (elephant shrew). For aquatic mammals we obtained the published sequences of four pinnipeds, nine cetaceans, the manatee and the sea otter. Finally, to ensure our phylogenetic trees included a range of branch lengths, we also obtained the published sequences of an additional 12 carnivores, three ungulates, ten rodents, one rabbit, five primates, three afrotherians, three marsupials and one prototherian. For details of taxa and accession numbers, see Table S1, Supplementary Material online.

### DNA Extraction and Sequencing

Genomic DNA was extracted from either muscle tissue or, for bats, wing membrane biopsies, using Qiagen DNeasy kits. The rhodopsin gene includes five exons interrupted by four introns. Three primer pairs were designed from conserved regions of primates, rodents, cow and dog and used to amplify three overlapping fragments (see Table S2, Supplementary Material online). For one taxon (Hottentot golden mole) these did not work and thus four additional primer pairs were used to amplify exon by exon (see Table S2, Supplementary Material online).

Polymerase Chain Reactions (PCR) contained 1 µl (50 ng/µl) genomic DNA, 5 µl 10 x buffer, 1.5 µl (50 mM) MgCl_2_, 1 µl (10 µM) of each primer and 1 U *Taq* DNA polymerase (Takara). Reactions were performed on a DNA Engine Dyad Cycler (BioRad) with the following conditions: initial denaturation step of 5 min; 30 cycles of denaturation at 94°C for 30 s, annealing temperature (see Table S2, Supplementary Material online) for 30 s; extension at 72°C for 30 to 180 s (depending upon the target length), and a final extension of 72°C for 5 min. PCR products were checked on an agarose gel and cloned into a pMD19-T vector (Takara). Positive clones were sequenced on an ABI sequencer using the sequencing primer pair M13–47 and M13–48 (see Table S2, Supplementary Material online). In order to avoid artifacts, multiple clones of each PCR product were sequenced in both forward and reverse directions.

### RNA Extraction and Sequencing

To verify the coding sequences, we amplified mRNA from the retinal tissue of two non-echolocating fruit bats (*Eonycteris spelaea* and *Rousettus leschenaultii*) two high-duty-cycle bats (*Rhinolophus ferrumequinum* and *Hipposideros pratti*) and three low-duty-cycle bats (*Taphozous melanopogon*, *Chaerephon plicatus* and *Myotis ricketti*). All of these individuals were collected from China and euthanized as part of a previous project for investigating the animal reservoir of SARS-CoV and in accordance with the guidelines of the China Practice for the Care and Use of Laboratory Animals. Eyes were stored in liquid nitrogen and total RNA isolated using TRIZOL (Invitrogen). First-strand synthesis of cDNA was undertaken using SuperScriptTM II reverse transcriptase (Invitrogen). PCRs mixture included 1 µg of the first-strand cDNA, 0.2 µM of the primers RHFc and RHRc (see Table S2, Supplementary Material online) and 1 U *Taq* DNA polymerase (Takara). This yielded a target length of ∼1.1 kb. PCR conditions and cloning protocols were the same as those used for genomic DNA.

### Sequence Alignment and Phylogenetic Analysis

For genomic DNA, intron-exon boundaries were identified from conserved splice signals (GT/AG) and, where possible, by comparison with published cDNA sequences. Sequences were aligned using CLUSTALX 1.81 [57], and checked by eye. We obtained >90% of continuous coding sequence for each species we examined.

For phylogenetic reconstruction based on coding sequences, we estimated the best-fit model of sequence evolution to be HKY + I + G (base frequencies  = 0.2130, 0.3327, 0.4543; proportion of invariable sites  = 0.4169; gamma distribution shape parameter  = 0.8145) based on the AIC in Modeltest 3.7 [58]. Maximum likelihood (ML) and Bayesian approaches were undertaken to recover the rhodopsin phylogeny using PAUP* 4.10b [59] and MrBayes 3.1.1 [60], respectively. The ML tree was generated by using tree bisection and reconnection (TBR) branch swapping, and the ML bootstrap values were calculated from 100 ML replicate trees using nearest-neighbor interchange (NNI) method. Each bootstrap replicate was started with an initial tree via the neighbour-joining (NJ) method. For the Bayesian tree, we ran six simultaneous Markov chains for one million generations. We used a flat prior and discarded the first 300,000 generations as burn-in to ensure sampling at stationarity. We included seven outgroups: three birds (*Anas platyrhynchos*, AF021240; *Gallus gallus*, NM_001030606; *Taeniopygia guttata*, NM_001076695), two amphibians (*Xenopus tropicalis*, U59922; *Bufo marinus*, NM_001097334) and two reptiles (*Alligator mississippiensis*, U23802; *Ambystoma tigrinum*, U36574).

To test for a significant difference between the published species tree and our gene tree [34], [35], we undertook Shimodaria–Hasgawa (SH) tests [61] in PAUP* 4.10b, with full optimization (two-tailed) and RELL bootstrap (one-tailed), respectively. These tests were calculated with 1,000 bootstrap replicates. We then repeated this test separately comparing the published species tree with gene tree in which we either (a) constrained the clade Rodentia or (b) constrained the clade Chiroptera (bats).

### Ancestral Reconstruction of Critical Sites

We reconstructed the ancestral states of the critical sites (wave-length specific sites) that control spectral tuning of rhodopsin using two methods. First, after removing the incomplete sequences of the horse and Hottentot golden mole, we used the maximum likelihood method [62] implemented in the PAML package. This approach calculates both the joint and marginal ancestral reconstructions. The former seeks to find the most likely character for all internal nodes, which maximizes the joint likelihood of the tree, whereas the latter compares the likelihood of all possible amino acids at a particular interior node and selects the one that yields the maximum likelihood tree. Second, we also used the parsimony approach in Mesquite version 2.6 [63], which generates the ancestral states that minimize the number of evolutionary steps. Since the parsimony method allows missing data, horse and Hottentot golden mole were included. We modeled each nucleotide as one character, and reconstructed the ancestral states at each node for each character, then checked the positions where the critical sites are located.

### Tests for Selection

To determine whether the rhodopsin gene has undergone accelerated evolution in mammal species that are adapted to low light conditions, we derived maximum-likelihood estimates of the rate of non-synonymous substitutions (d_N_) and the rate of synonymous substitutions (d_S_) using the CODEML program in PAML version 4 [64]. The ratio d_N_/d_S_, termed omega (ω), is <1 where purifying selection dominates, approximates to 1 where neutral evolution dominates and is >1 when positive selection dominates. We used an unrooted tree based on the results of our phylogenetic reconstruction (Figure 1) following the removal of the non-mammalian outgroups. Where the gene tree differed from the species tree, we repeated the analyses with the species tree topology. Since differences in estimates of substitution rates will be influenced by species coverage, we also repeated our analyses with a reduced dataset comprising just bats.

For each dataset, we modeled selection using a combination of branch models, site models and clade models. For branch models, we first estimated an independent ω value for each branch under the free-ratio model. Second, we estimated ω under a one-ratio model in which the same ratio occurs across the tree, and third, we used the two-ratio ‘branch model’ to compare the estimated ω ratio on specific foreground branches (ω_1_) in the phylogeny to the background ratio (ω_0_) (Figure 1 and Table S3, Supplementary Material online). Branch models were applied to branches leading to taxa or clades of taxa that are adapted for living in low light environments, several of which also show loss of their *SWS1* opsin gene.

Two pairs of sites models were implemented. The nearly neutral model (M1a) assumes two classes of sites: one is under purifying selection with 0<ω_0_<1, the other is under neutral evolution with ω_1_ = 1, and was compared to the positive selection model (M2a) in which an additional ω parameter is included that allows positive selection where present (ω>1). We also used the M8a model (β & ω = 1) which constrains 0<ω<1 over sites following a β distribution and allows ω = 1 at some sites, and compared this to M8 model (β & ω model), in which positive selection is allowed.

Finally, we tested whether ω was on average higher in groups of related key taxa than in the background tree by implementing Clade Model C, which includes three site classes. Classes 0 and 1 represent purifying selection (0<ω_0_<1) and neutral evolution (ω_1_ = 1), respectively, and are assumed to be shared between the focal clade and the background, whereas the selection pressure at the third site class can differ between the clade and background (*ω*_2_≠*ω*_3_).

Significant model improvement was assessed using likelihood ratio tests (LRT) to compare nested models. To test for heterogeneous selection pressure across the tree, we compared the free-ratio and one-ratio branch models, and to test for positive selection on focal branches we compared the one and two-ratio branch models. For sites modes, we tested for positive selection by comparing M1a versus M2a, and M8a versus M8. Finally, Clade Model C was compared to M1a to detect divergent selection acting on groups of related taxa.

### Sliding Window Analysis

To explore further the heterogeneous selection pressure across the rhodopsin gene, we constructed a sliding window of ω values estimated using the Nei and Gojobori method [65]. Sliding windows, which were repeated for several groups of interest, were implemented in the program SWAAP 1.0.2 [66] with window and step sizes of 30 and 3 bp nucleotides, respectively.

## Supporting Information

Figure S1 An alignment of deduced amino acids of the rhodopsin genes sequenced in this study (only the variable sites shown). Amino acid positions given above the alignment correspond to the complete rhodopsin gene of cow (*Bos taurus*) and sites identical to the cow sequence were indicated with a dot (.). Missing data were showed with a dash (-).
(0.12 MB PDF)

Figure S2 The species tree showing the inferred rhodopsin wavelengths of maximum absorption (λ_max_) for extant and ancestral taxa. A question mark (?) indicates that the λ_max_ was unable to be inferred on the basis of the current data. Critical amino acid substitutions are given above the branches.
(0.28 MB PDF)

Table S1 Taxa used in the study
(0.03 MB PDF)

Table S2 Primers used in this study
(0.01 MB PDF)

Table S3 Likelihood values and parameter estimates for mammalian rhodopsin genes
(0.03 MB PDF)

Table S4 Summary of the 13 key amino acid sites for rhodopsins
(0.04 MB PDF)
