# Supplementary figures and images for: Rhodopsin Molecular Evolution in Mammals Inhabiting Low Light Environments
Source: PLoS One. 2009 Dec 16;4(12):e8326. doi: 10.1371/journal.pone.0008326 (PMC2790605; doi:10.1371/journal.pone.0008326)

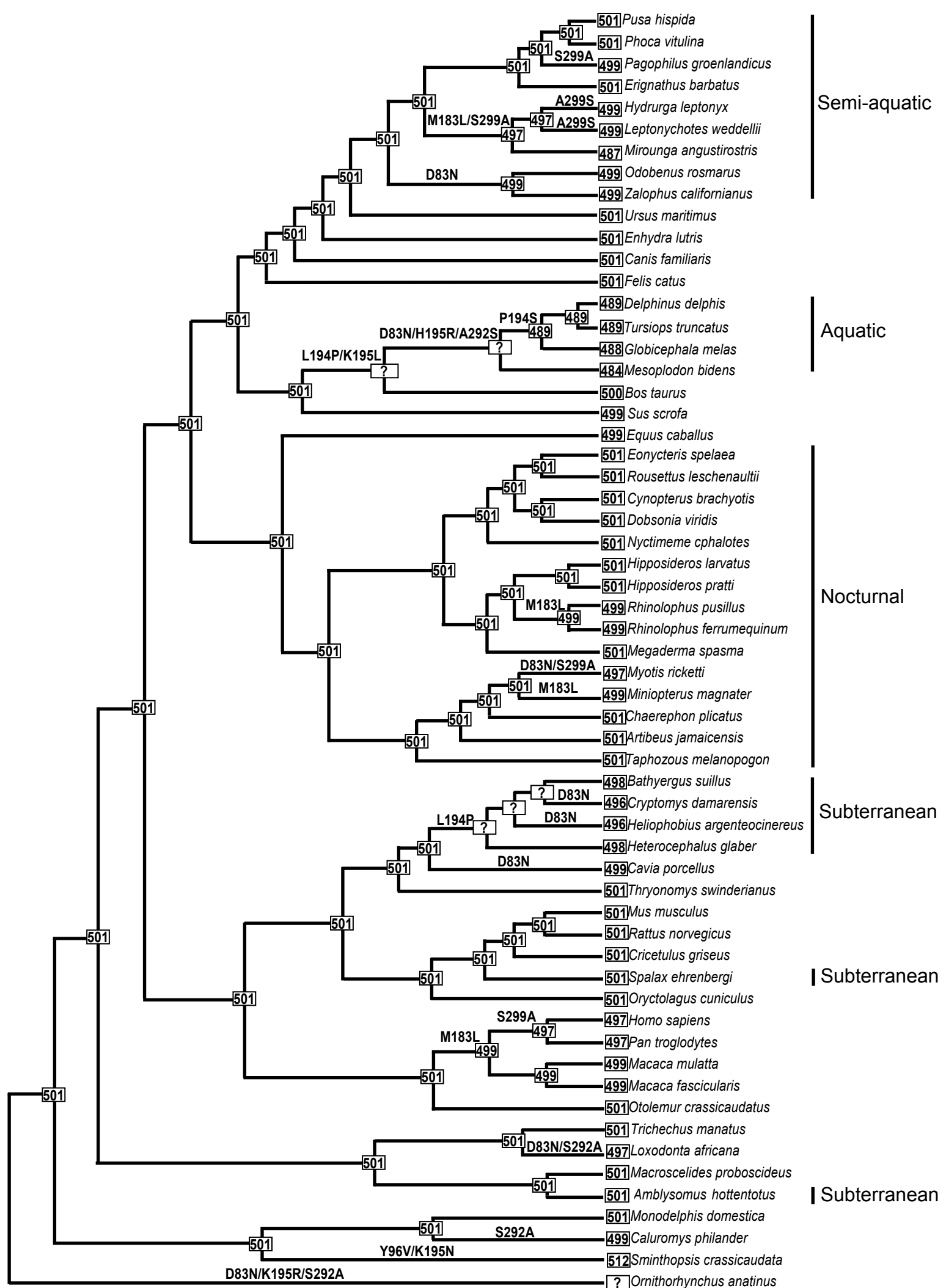

Supplement: Figure S2 — The species tree showing the inferred rhodopsin wavelengths of maximum absorption (λmax) for extant and ancestral taxa. A question mark (?) indicates that the λmax was unable to be inferred on the basis of the current data. Critical amino acid substitutions are given above the branches. (0.28 MB PDF) [file pone.0008326.s002.pdf]
