# Supplementary material for: Rhodopsin Molecular Evolution in Mammals Inhabiting Low Light Environments
Source: PLoS One. 2009 Dec 16;4(12):e8326. doi: 10.1371/journal.pone.0008326 (PMC2790605; doi:10.1371/journal.pone.0008326)
Supplement: Table S1 — Taxa used in the study (0.03 MB PDF) [file pone.0008326.s003.pdf]

Supplementary Table S1. Classification of taxa and GenBank accession numbers for rhodopsin gene in this study. Accession numbers of new sequences are shown in bold.

Classification			Common name	Species name	Acc. No
Boreoeutheria	Laurasiatheria	ORDER CHIROPTERA (BATS)			
		SUBORDER YINPTEROCHIROPTERA			
		Family Pteropodidae	lesser dawn bat	*Eonycteris spelaea*	**GQ290305**
			Leschenault's rousette	*Rousettus leschenaultii*	**GQ290317**
			Pallas's tube-nosed fruit bat	*Nyctimene cephalotes*	**GQ290313**
			greenish naked-backed fruit bat	*Dobsonia viridis*	**GQ290304**
			lesser short-nosed fruit bat	*Cynopterus brachyotis*	**GQ290303**
		Family Megadermatidae	lesser false vampire bat	*Megaderma spasma*	**GQ290316**
		Family Rhinolophidae	least horseshoe bat	*Rhinolophus pusillus*	**GQ290315**
			greater horseshoe bat	*Rhinolophus ferrumequinum*	**GQ290314**
			Pratt's leaf-nosed bat	*Hipposideros pratti*	**GQ290309**
			intermediate leaf-nosed bat	*Hipposideros larvatus*	**GQ290308**
		SUBORDER YANGOCHIROPTERA			
		Family Emballonuridae	black-bearded tomb bat	*Taphozous melanopogon*	**GQ290318**
		Family Phyllostomidae	Jamaican fruit-eating bat	*Artibeus jamaicensis*	**GQ290299**
		Family Miniopteridae	western long-fingered bat	*Miniopterus fuliginosus*	**GQ290311**
		Family Molossidae	wrinkle-lipped free-tailed bat	*Chaerephon plicatus*	**GQ290301**
		Family Vespertilionidae	Rickett's big-footed myotis	*Myotis ricketti*	**GQ290312**
		ORDER CARNIVORA			
		Family Canidae	domestic dog	*Canis lupus*	X71380
		Family Felidae	domestic cat	*Felis catus*	NM_001009 242
		Family Phocidae	harp seal	*Pagophilus groenlandicus*	AF055318
			harbor seal	*Phoca vitulina*	AF055317
			ringed seal	*Pusa hispida*	AY883927
			bearded seal	*Erignathus barbatus*	AY883932
			leopard seal	*Hydrurga leptonyx*	AY883930
			Weddell seal	*Leptonychotes weddellii*	AY883929
			Northern elephant seal	*Mirounga angustirostris*	AY883928
		Family Odobenidae	walrus	*Odobenus rosmarus*	AY883925
		Family Otariidae	California sealion	*Zalophus californianus*	AY883924
		Family Mustelidae	sea otter	*Enhydra lutris*	AY883931
		Family Ursidae	polar bear	*Ursus maritimus*	AY883926
		ORDER PERISSODACTYLA			
		Family Equidae	horse	*Equus caballus*	XM_001490 301
		ORDER CETARTIODACTYLA			
		Family Bovidae	cow	*Bos taurus*	NM_001014 890
			wild boar	*Sus scrofa*	NM_214221
		ORDER CETACEA			
		Family Delphinidae	bottlenose dolphin	*Tursiops truncatus*	AF055456
			short-beaked common dolphin	*Delphinus delphis*	AF055314
			long-finned pilot whale	*Globicephala melas*	AF055315
		Family Ziphiidae	Sowerby's beaked whale	*Mesoplodon bidens*	AF055316
	Euarchontoglires	ORDER RODENTS			
		SUBORDER MYOMORPHA			
		Family Muridae	house mouse	*Mus musculus*	NM_145383
			brown rat	*Rattus norvegicus*	NM_033441
		Family Cricetidae	striped dwarf hamster	*Cricetulus barabensis*	X61084
		Family Spalacidae	Middle East blind mole rat	*Spalax ehrenbergi*	AF309568

Supplementary Table S1 continued

		SUBORDER HYSTRICOMORPHA			
		Family Thryonomyidae	greater cane rat	*Thryonomys swinderianus*	**GQ290319**
		Family Bathyergidae	naked mole-rat	*Heterocephalus glaber*	**GQ290307**
			silvery mole-rat	*Heliophobius argenteocinereus*	**GQ290306**
			Damaraland mole-rat	*Cryptomys damarensis*	**GQ290302**
			cape dune mole rat	*Bathyergus suillus*	**GQ290300**
		Family Caviidae	domesticated Guinea pig	*Cavia porcellus*	EF457995
		ORDER LAGOMORPHA			
		Family Leporidae	European rabbit	*Oryctolagus cuniculus*	NM_001082 349
		ORDER PRIMATES			
		Family Cercopithecidae	crab-eating macaque	*Macaca fascicularis*	S76579
			rhesus monkey	*Macaca mulatta*	XM_001094 250
		Family Hominidae	human	*Homo sapiens*	NM_000539
			common chimpanzee	*Pan troglodytes*	XM_516740
		Family Galagidae	brown greater galago	*Otolemur crassicaudatus*	AB112594
Atlantogenata	Afrotheria	ORDER PROBOSCIDEA			
		Family Elephantidae	African bush elephant	*Loxodonta africana*	AY686752
		ORDER SIRENIA			
		Family Trichechidae	West Indian manatee	*Trichechus manatus*	AF055319
		ORDER MACROSCELIDEA			
		Family Macroscelididae	short-eared elephant shrew	*Macroscelides proboscideus*	**GQ290310**
		ORDER AFROSORICIDA			
		Family Chrysochloridae	Hottentot golden mole	*Amblysomus hottentotus*	**GQ290298**
Marsupialia		ORDER DASYUROMORPHIA			
		Family Dasyuridae	fat-tailed dunnart	*Sminthopsis crassicaudata*	AY159786
		ORDER DIDELPHIMORPHIA			
		Family Didelphidae	gray short-tailed opossum	*Monodelphis domestica*	XM_001366 188
			bare-tailed woolly opossum	*Caluromys philander*	AY313946
Prototheria		ORDER MONOTREMATA			
		Family Ornithorhynchidae	platypus	*Ornithorhynchus anatinus*	NM_001127 627
