# Supplementary material for: Rhodopsin Molecular Evolution in Mammals Inhabiting Low Light Environments
Source: PLoS One. 2009 Dec 16;4(12):e8326. doi: 10.1371/journal.pone.0008326 (PMC2790605; doi:10.1371/journal.pone.0008326)
Supplement: Table S2 — Primers used in this study (0.01 MB PDF) [file pone.0008326.s004.pdf]

Supplementary Table S2. Primers used in this study

Primer name	Primer sequence (5' to 3')[1]	Ta (°C)	Product
RHFc[2]	CC[G/A]CAGCCATGAA[C/T]GG[G/C]AC[G/A]GA[G/A]GGCCC[G/T]AACTTCTAC	55-60°C	Exon 1-Exon 2 (~2400bp)
RDaR[2]	GC[G/A]CAGGCCAG[C/T]GCCATGACCCAGG		
RDbF[2]	AGGTGAAATTGCCCTGTGGTCCTTGGTGGTC	55-60°C	Exon 2-Exon 3 (~1500bp)
RDbR[2]	GGGATGGTGAAGTGGACCAC[G/A]AACATGTAGA		
RDcF[2]	GGCATGCAGTGCTC[A/G]TG[T/C]GGGAT[C/T]GAC	55-60°C	Exon 3-Exon 5 (~1400bp)
RHRc[2]	GG[G/T]CTTAGGC[C/T]GG[T/G]GCCACCTGGCT[G/C]GTCTCCGTCTTG		
G5F[3]	AGCCATGAA[C/T]GG[G/C]ACAGAAG	50-55°C	Exon 1 (~370bp)
G5R[3]	C[A/G]GCTCATACC[G/T]CCCAGGGTG		
H2F[3]	T[G/A] CAGGTGAAAT[T/C]GCCCT[G/A]TGG	50-55°C	Exon 2 (~200bp)
H1R[3]	CAGTGCCATTACCTGGACCA		
J2F[3]	TCAGGTA[C/T]AT[C/T]CC[A/G]GAAG	50-55°C	Exon 3-Exon 4 (~550bp)
J2R[3]	GGCACCTGCTTATTCATC		
K2F[3]	C[T/C][C/T]TTGCCTTCCAGTTCCG	50-55°C	Exon 5 (~150bp)
K1R[3]	CACCTGGCT[C/G]GTCTCCGTCTTG		
Sequencing primer pair			
M13-47[4]	CGCCAGGGTTTTCCCAGTCACGAC	55°C	
M13-48[4]	GAGCGGATA ACA ATTTCACACAGG		

[1] Start and stop codons are underlined
[2] Primer used in all mammals
[3] Primer used in Hottentot golden mole only
[4] Primer used in sequencing
