# Supplementary material for: Rhodopsin Molecular Evolution in Mammals Inhabiting Low Light Environments
Source: PLoS One. 2009 Dec 16;4(12):e8326. doi: 10.1371/journal.pone.0008326 (PMC2790605; doi:10.1371/journal.pone.0008326)
Supplement: Table S3 — Likelihood values and parameter estimates for mammalian rhodopsin genes (0.03 MB PDF) [file pone.0008326.s005.pdf]

Supplementary table S3. Likelihood values and parameter estimates for mammalian rhodopsin genes

| Model code (a) | $\ell$ (b) | *P* (c) | Parameter estimates |
|---|---|---|---|
| Dataset I: all mammals | | | |
| *Branch Models* | | | |
| A1: One-ratio | -9008.20 | 118 | $\omega$=0.040 |
| B1: Free-ratio | -8843.40 | 233 | variable $\omega$ by branch |
| C1: Two-ratio: AFM branch, background | -9006.28 | 119 | $\omega_0$=0.039, $\omega_1$=0.104 |
| D1: Two-ratio: Middle East blind mole rat branch, background | -9007.92 | 119 | $\omega_0$=0.040, $\omega_1$=0.055 |
| E1: Two-ratio: Hottentot golden mole branch, background | -9008.20 | 119 | $\omega_0$=0.040, $\omega_1$=0.038 |
| F1: Two-ratio: CET branch, background | -9004.02 | 119 | $\omega_0$=0.039, $\omega_1$=0.202 |
| G1: Two-ratio: PIN branch, background | -9007.87 | 119 | $\omega_0$=0.040, $\omega_1$=0.000 |
| H1: Two-ratio: West Indian manatee branch, background | -9005.33 | 119 | $\omega_0$=0.041, $\omega_1$=0.009 |
| I1: Two-ratio: sea otter branch, background | -9008.15 | 119 | $\omega_0$=0.040, $\omega_1$=0.034 |
| J1: Two-ratio: BAT branch, background | -9008.20 | 119 | $\omega_0$=0.040, $\omega_1$=0.038 |
| *Site Models* | | | |
| K1: M1a: nearly neutral | -8857.65 | 119 | $\rho_0$=0.936 ($\rho_1$=0.064)<br>$\omega_0$=0.025 ($\omega_1$=1.000) |
| L1: M2a: positive selection | -8857.65 | 121 | $\rho_0$=0936, $\rho_1$=0.064 ($\rho_2$=0.000)<br>$\omega_0$=0.025, $\omega_1$=1.000 ($\omega_2$=75.307) |
| M1: M8a: $\beta$ & $\omega$=1 | -8663.43 | 119 | p=0.127, q=1.984 |
| N1: M8: $\beta$ & $\omega$ | -8663.44 | 121 | $\rho_0$=1.000, p=0.127, q=1.984<br>$\rho_1$=0.000, $\omega$=2.724 |
| *Clade Models* | | | |
| O1: Model C: all African mole-rats | -8676.08 | 122 | $\omega_0$=0.005, $\rho_0$=0.816; $\omega_1$=1, $\rho_1$=0.010<br>$\omega_2$=0.199, $\omega_3$=0.298 ($\rho_2$=0.174) |
| P1: Model C: all pinnipeds | -8658.42 | 122 | $\omega_0$=0.006, $\rho_0$=0.820; $\omega_1$=1, $\rho_1$=0.004<br>$\omega_2$=0.197, $\omega_3$=1.262 ($\rho_2$=0.177) |
| Q1: Model C: all cetaceans | -8660.95 | 122 | $\omega_0$=0.005, $\rho_0$=0.811; $\omega_1$=1, $\rho_1$=0.015<br>$\omega_2$=0.180, $\omega_3$=1.205 ($\rho_2$=0.174) |
| R1: Model C: all bats | -8668.22 | 122 | $\omega_0$=0.005, $\rho_0$=0.815; $\omega_1$=1, $\rho_1$=0.007<br>$\omega_2$=0.236, $\omega_3$=0.102 ($\rho_2$=0.178) |
| Dataset II: bats only | | | |
| *Branch Models* | | | |
| A2: One-ratio | -2963.02 | 29 | $\omega$=0.023 |
| B2: Free-ratio | -2945.07 | 55 | variable $\omega$ by branch |
| C2: Two-ratio: OWF branch, background | -2962,76 | 30 | $\omega_0$=0.022, $\omega_1$=0.035 |
| D2: Two-ratio: HDC branch, background | -2961.85 | 30 | $\omega_0$=0.022, $\omega_1$= $\infty$ |
| E2: Two-ratio: YAN branch, background | -2960.73 | 30 | $\omega_0$=0.024, $\omega_1$= 0.001 |
| *Site Models* | | | |
| F2: M1a: nearly neutral | -2954.28 | 30 | $\rho_0$=0.978 ($\rho$1=0.022)<br>$\omega_0$=0.015 ($\omega_1$=1.000) |
| G2: M2a: positive selection | -2954.29 | 32 | $\rho_0$=0.978, $\rho_1$=0.022 ($\rho_2$=0.000)<br>$\omega_0$=0.015, $\omega_1$=1.000 ($\omega_2$=45.178) |
| H2: M8a: $\beta$ & $\omega$=1 | -2949.45 | 30 | p=0.101, q=3.231 |
| I2: M8: $\beta$ & $\omega$ | -2949.45 | 32 | $\rho_0$=1.000, p=0.101, q=3.231<br>$\rho_1$=0, $\omega$=1.000 |
| *Clade Models* | | | |
| J2: Model C: all Old World fruit bats | -2948.75 | 33 | $\omega_0$=0.009, $\rho_0$=0.936; $\omega_1$=1, $\rho_1$=0<br>$\omega_2$=0.297, $\omega_3$=0.267 ($\rho_2$=0.064) |
| K2: Model C: all high-duty-cycle bats | -2948.73 | 33 | $\omega_0$=0.009, $\rho_0$=0.935; $\omega_1$=1, $\rho_1$=0<br>$\omega_2$=0.273, $\omega_3$=0.339 ($\rho_2$=0.065) |
| L2: Model C: all Yangochiroptera | -2948.53 | 33 | $\omega_0$=0.009, $\rho_0$=0.944; $\omega_1$=1, $\rho_1$=0<br>$\omega_2$=0.368, $\omega_3$=0.263 ($\rho_2$=0.056) |

(a) Codes for focal branches (e.g. AFM) are given in fig. 1; (b) Likelihood values; (c) Number of parameters
