# Supplementary material for: Rhodopsin Molecular Evolution in Mammals Inhabiting Low Light Environments
Source: PLoS One. 2009 Dec 16;4(12):e8326. doi: 10.1371/journal.pone.0008326 (PMC2790605; doi:10.1371/journal.pone.0008326)
Supplement: Table S4 — Summary of the 13 key amino acid sites for rhodopsins (0.04 MB PDF) [file pone.0008326.s006.pdf]

Table S4. Summary of the 13 key amino acid sites for rhodopsins. For λ_{max} values, those predicted from this study are given in bold and those from the literature are in plain text. Amino acids identical to the mammalian consensus are indicated by a point (.), n/a represents missing data.

Species	83	96	102	122	183	194	195	253	261	289	292	299	317	λ_{max} (measured)	λ_{max} (predicted)
Consensus	D	Y	Y	E	M	L	K	M	F	T	A	S	M		
Hipposideros larvatus	.	.	.	.	.	.	.	.	.	.	.	.	.		**501**
Hipposideros pratti	.	.	.	.	.	.	.	.	.	.	.	.	.		**501**
Rhinolophus pusillus	.	.	.	.	L	.	.	.	.	.	.	.	.		**499**
Rhinolophus ferrumequinum	.	.	.	.	L	.	.	.	.	.	.	.	.		**499**
Megaderma spasma	.	.	.	.	.	.	.	.	.	.	.	.	.		**501**
Eonycteris spelaea	.	.	.	.	.	.	.	.	.	.	.	.	.		**501**
Rousettus leschenaultii	.	.	.	.	.	.	.	.	.	.	.	.	.		**501**
Nyctimeme cphalotes	.	.	.	.	.	.	.	.	.	.	.	.	.		**501**
Dobsonia viridis	.	.	.	.	.	.	.	.	.	.	.	.	.		**501**
Cynopterus brachyotis	.	.	.	.	.	.	.	.	.	.	.	.	.		**501**
Myotis ricketti	N	.	.	.	.	.	.	.	.	.	.	A	.		**497**
Taphozous melanopogon	.	.	.	.	.	.	.	.	.	.	.	.	.		**501**
Miniopterus fuliginosus	.	.	.	.	L	.	.	.	.	.	.	.	.		**499**
Chaerephon plicatus	.	.	.	.	.	.	.	.	.	.	.	.	.		**501**
Artibeus jamaicensis	.	.	.	.	.	.	.	.	.	.	.	.	.		**501**
Pusa hispida	.	.	.	.	.	.	.	.	.	.	.	.	.		501[a]
Phoca vitulina	.	.	.	.	.	.	.	.	.	.	.	.	.	501[b]	501[a]
Pagophilus groenlandica	.	.	.	.	.	.	.	.	.	.	.	A	.	498[b]	499[a]
Erignathus barbatus	.	.	.	.	.	.	.	.	.	.	.	.	.		501[a]
Hydrurga leptonyx	.	.	.	.	L	.	.	.	.	.	.	.	.		499[a]
Leptonychotes weddellii	.	.	.	.	L	.	.	.	.	.	.	.	.	495, 496[c]	499[a]
Mirounga angustirostris	.	.	.	.	L	.	.	.	.	.	S	.	.	483[d]	487[a]
Odobenus rosmarus	N	.	.	.	.	.	.	.	.	.	.	.	.		499[a]
Zalophus californianus	N	.	.	.	.	.	.	.	.	.	.	.	.	499[a]	499[e]
Ursus maritimus	.	.	.	.	.	.	.	.	.	.	.	.	.		501[a]
Enhydra lutris	.	.	.	.	.	.	N	.	.	.	.	.	.		501 [a]
Canis lupus	.	.	.	.	.	.	.	.	.	.	..	.	.	508[f]	501 [a]
Felis catus	.	.	.	.	.	.	.	.	.	.	.	.	.		501 [a]
Delphinus delphis	N	.	.	.	.	.	S	.	.	.	S	.	.	489[g]	
Tursiops truncatus	N	.	.	.	.	S	R	.	.	.	S	.	.	488, 489 [g]	
Globicephala melas	N	.	.	.	.	S	R	.	.	.	S	.	.	488 [g]	
Mesoplodon bidens	N	.	.	.	.	P	S	.	.	.	S	A	.	484 [g]	485[a]
Equus caballus	.	.	.	.	.	.	.	.	.	.	.	A	n/a		**499**
Bos taurus	.	.	.	.	.	P	H	.	.	.	.	A	.	500[h]	

Table S4 continued

Species	83	96	102	122	183	194	195	253	261	289	292	299	317	λ_{max} (measured)	λ_{max} (predicted)
Sus scrofa	.	.	.	.	L	.	.	.	.	.	.	.	.		**499**
Homo sapiens	.	.	.	.	L	.	.	.	.	.	.	A	.	497[i]	
Pan troglodytes	.	.	.	.	L	.	.	.	.	.	.	A	.		**497**
Macaca fascicularis	.	.	.	.	L	.	.	.	.	.	.	.	.	500[j]	**499**
Macaca mulatta	.	.	.	.	L	.	.	.	.	.	.	.	.		**499**
Otolemur crassicaudatus	.	.	.	.	.	.	.	.	.	.	.	.	.		**501**
Loxodonta africana	N	.	.	.	.	.	.	.	.	.	.	A	.	496[k]	**497**
Trichechus manatus	.	.	.	.	.	.	.	.	.	.	.	.	.	502[g]	**501**
Macroscelides proboscideus	.	.	.	.	.	.	.	.	.	.	.	.	.		**501**
Amblysomus hottentotus	.	.	.	n/a	.	.	.	.	.	.	.	.	.		**501**
Oryctolagus cuniculus	.	.	.	.	.	.	.	.	.	.	.	.	.	502[f]	**501**
Bathyergus suillus	.	.	.	.	.	P	.	.	.	.	.	.	.		**498**
Cryptomys damarensis	N	.	.	.	.	P	.	.	.	.	.	.	.		**496**
Heliophobius argenteocinereus	N	.	.	.	.	P	.	.	.	.	.	.	.		**496**
Heterocephalus glaber	.	.	.	.	.	P	.	.	.	.	.	.	.		**498**
Cavia porcellus	N	.	.	.	.	.	.	.	.	.	.	.	.		**499**
Thryonomys swinderianus	.	.	.	.	.	.	.	.	.	.	.	.	.		**501**
Rattus norvegicus	.	.	.	.	.	.	.	.	.	.	.	.	.	498 [f]	**501**
Mus musculus	.	.	.	.	.	.	.	.	.	.	.	.	.	498 [f]	**501**
Cricetulus griseus	.	.	.	.	.	.	.	.	.	.	.	.	.		**501**
Spalax ehrenbergi	.	.	.	.	.	.	.	.	.	.	.	.	.		**501**
Caluromys philander	.	.	.	.	.	.	.	.	.	.	.	A	.		**499**
Monodelphis domestica	.	.	.	.	.	.	.	.	.	.	.	.	.		**501**
Sminthopsis crassicaudata	.	V	.	.	.	.	N	.	.	.	.	.	.	512[l]	
Ornithorhynchus anatinus	N	.	.	.	.	.	R	.	.	..	.	A	.		

a) Levenson DH, Ponganis PJ, Crognale MA, Deegan JF II, Dizon A et al. (2006) Visual pigments of marine carnivores: pinnipeds, polar bear, and sea otter. J Comp Physiol A 192:833-843.
b) Fasick JI, Robinson PR (2000) Spectral-tuning mechanisms of marine mammal rhodopsins and correlations with foraging depth. Vis Neurosci 17: 781-788.
c) Lythgoe JN, Dartnall HJA (1970) A "deep sea rhodopsin" in a mammal. Nature 227:955-956.
d) Southall KD, Oliver GW, Lewis JW, Le Boeuf BJ, Levenson DH et al. (2002) Visual pigment sensitivity in three deep diving marine mammals. Mar Mam Sci 18:275-281.
e) Collins FD, Morton RA (1950). Studies on rhodopsin. I. Methods of extraction and the absorption spectrum. Biochem J 47:3-10.
f) Bridges CDB (1959) The visual pigments of some common laboratory mammals. Nature 184:1727-1728.
g) Oprian DD, Molday RS, Kaufman RJ, Khorana HG (1987). Expression of a synthetic bovine rhodopsin gene in monkey kidney cells. Proc Natl Acad Sci USA 84:8874-8878.
h) Crescitelli F, Dartnall HJ (1953). Human visual purple. Nature 172:195-197.
i) Bowmaker JK, Dartnall HJ, Lythgoe JN, Mollon JD (1978). The visual pigments of rods and cones in the rhesus monkey, *Macaca mulatta*. J Physiol 274:329-348.
j) Yokoyama S, Takenaka N, Agnew DW, Shoshani J (2005) Elephants and human color-blind deuteranopes have identical sets of visual pigments. Genetics 170:335-344.
k) Hunt DM, Arrese CA, von Dornum M, Rodger J, Oddy A et al. (2003) The rod opsin pigments from two marsupial species, the South American bare-tailed woolly opossum and the Australian fat-tailed dunnart. Gene 323:157-162.
