# Supplementary material for: Rhodopsin Molecular Evolution in Mammals Inhabiting Low Light Environments
Source: PLoS One. 2009 Dec 16;4(12):e8326. doi: 10.1371/journal.pone.0008326 (PMC2790605; doi:10.1371/journal.pone.0008326)
Supplement: Figure S1 — An alignment of deduced amino acids of the rhodopsin genes sequenced in this study (only the variable sites shown). Amino acid positions given above the alignment correspond to the complete rhodopsin gene of cow (Bos taurus) and sites identical to the cow sequence were indicated with a dot (.). Missing data were showed with a dash (-). (0.12 MB PDF) [file pone.0008326.s001.pdf]

	11	16	19	26	46	49	54	83	88	89	99	102	104	107	112	123	137	154	155	157	158	159	162	165	169	173
Hipposideros larvatus	V	K	V	Y	L	V	I	D	F	G	L	Y	I	P	L	I	V	I	M	L	A	F	V	L	A	A
Hipposideros pratti	.	.	.	.	.	.	.	.	.	.	.	.	.	.	.	.	.	.	.	.	.	.	.	.	.	.
Rhinolophus pusillus	.	.	.	.	.	.	.	.	.	.	.	F	.	.	.	V	.	.	.	.	.	S	.	.	V	.
Rhinolophus ferrumequinum	.	.	.	.	.	.	.	.	.	.	.	F	.	.	.	V	.	.	.	.	.	S	.	.	.	.
Megaderma spasma	.	.	.	.	.	.	.	.	.	.	.	.	V	.	.	.	.	.	.	V	.	.	.	.	.	.
Eonycteris spelaea	.	.	.	.	.	.	.	.	.	.	.	.	V	.	.	.	.	.	.	.	.	L	.	.	.	V
Rousettus leschenaultii	.	.	.	.	.	.	.	.	.	.	.	.	V	.	.	.	.	.	.	.	.	L	.	.	.	V
Nyctimene cephalotes	.	.	.	H	.	.	.	.	.	.	.	.	V	.	.	.	.	.	.	.	.	L	.	.	.	V
Dobsonia viridis	.	.	.	H	.	.	.	.	.	.	.	.	V	.	.	.	.	.	.	.	.	L	.	.	.	V
Cynopterus brachyotis	.	.	.	H	.	.	.	.	.	.	.	.	V	.	.	.	.	.	.	.	.	L	.	.	.	V
Myotis ricketti	.	.	.	.	.	.	V	N	.	.	M	.	V	A	.	.	.	.	.	.	.	.	.	.	.	.
Taphozous melanopogon	.	.	.	.	.	.	.	.	.	.	M	.	V	.	.	.	.	.	.	V	.	.	.	.	.	.
Miniopterus fuliginosus	.	.	.	.	.	.	.	.	.	.	.	.	.	.	.	.	.	.	.	.	.	.	.	.	.	.
Chaerephon plicatus	.	.	.	H	.	.	.	.	.	.	.	.	V	.	.	V	.	.	.	.	.	.	.	.	.	.
Artibeus jamaicensis	.	.	.	.	.	.	.	.	.	.	.	.	V	.	.	.	.	.	.	.	V	C	I	.	.	.
Macroscelides proboscideus	I	.	.	.	M	.	.	.	L	.	.	.	V	.	V	.	.	.	.	V	.	.	I	M	.	.
Amblysomus hottentotus	.	Q	.	.	.	.	.	.	L	.	.	.	V	.	.	-	.	.	.	.	.	.	.	.	.	.
Bathyergus suillus	.	I	.	.	.	.	.	.	I	C	M	.	.	.	M	.	.	.	.	V	.	L	.	.	.	.
Cryptomys damarensis	.	I	.	.	.	.	.	N	I	C	M	.	V	.	M	.	M	V	.	V	.	.	.	.	.	.
Heliophobius argenteocinereus	.	I	.	.	.	M	V	N	I	C	M	.	V	.	M	.	.	.	.	V	T	.	.	.	.	.
Heterocephalus glaber	.	I	M	.	.	.	V	.	I	C	M	.	V	A	M	.	M	.	V	V	.	.	.	.	.	.
Thryonomys swinderianus	.	A	.	.	.	.	.	.	.	.	.	.	V	.	.	.	.	.	.	V	.	.	.	.	.	.

	194	197	198	199	216	217	218	256	259	263	266	270	273	277	282	286	290	292	297	298	299	321	326	327	328	337
Hipposideros larvatus	L	E	V	N	M	I	V	I	I	I	L	S	F	T	N	I	L	A	S	S	S	L	S	P	L	A
Hipposideros pratti	.	.	.	.	.	.	.	.	.	.	.	.	.	.	.	.	.	.	.	.	.	.	.	.	.	.
Rhinolophus pusillus	.	.	.	.	.	.	.	.	.	.	.	.	.	.	.	.	.	.	.	A	.	.	N	.	.	.
Rhinolophus ferrumequinum	.	.	.	.	.	.	.	.	.	.	.	.	.	.	.	.	.	.	.	A	.	.	N	.	.	.
Megaderma spasma	.	.	.	.	.	.	.	.	.	.	V	.	.	.	.	.	.	.	.	.	.	.	N	I	.	.
Eonycteris spelaea	.	.	.	.	.	.	.	.	.	.	.	G	.	.	.	.	.	.	.	.	.	.	N	.	.	.
Rousettus leschenaultii	.	.	.	.	.	.	.	.	.	.	.	G	.	.	.	.	.	.	.	.	.	.	N	.	.	.
Nyctimene cephalotes	.	.	.	.	.	.	.	.	.	.	.	G	.	.	.	.	.	.	.	.	.	.	N	.	.	.
Dobsonia viridis	.	.	.	.	.	.	.	.	.	.	.	G	.	.	.	.	.	.	T	.	.	.	N	.	.	.
Cynopterus brachyotis	.	.	.	.	.	.	.	.	.	.	.	G	.	.	.	.	.	.	.	.	.	.	N	.	.	.
Myotis ricketti	.	.	.	.	.	.	.	.	V	.	.	.	.	.	.	V	I	.	.	.	A	.	N	.	.	.
Taphozous melanopogon	.	.	.	.	.	.	.	.	.	.	V	G	.	.	.	.	.	.	.	.	.	.	N	.	.	.
Miniopterus fuliginosus	.	.	.	.	.	.	.	.	.	.	.	.	.	.	.	.	.	.	.	.	.	.	N	.	.	.
Chaerephon plicatus	.	.	.	.	.	V	.	.	.	.	.	.	.	S	D	.	.	.	.	.	.	.	N	.	.	.
Artibeus jamaicensis	.	.	.	.	.	.	.	.	.	.	.	.	.	.	.	.	.	.	.	.	.	.	N	.	.	.
Macroscelides proboscideus	.	.	.	.	L	V	I	.	.	.	V	.	I	.	.	.	I	.	.	.	.	.	N	.	F	V
Amblysomus hottentotus	.	D	I	S	.	V	.	L	.	V	.	G	L	.	.	.	.	.	.	A	.	I	.	L	F	V
Bathyergus suillus	P	.	I	.	L	V	I	.	.	.	V	.	V	.	D	.	I	.	.	.	.	I	N	.	.	.
Cryptomys damarensis	P	.	I	.	L	V	I	.	.	.	V	.	V	.	D	.	I	.	.	.	.	I	N	.	.	.
Heliophobius argenteocinereus	P	.	I	.	L	V	I	.	.	.	V	.	V	.	D	.	I	S	.	.	.	I	N	.	.	.
Heterocephalus glaber	P	.	I	.	L	V	I	.	.	.	V	.	M	.	D	.	I	.	.	.	.	I	.	.	.	.
Thryonomys swinderianus	.	.	.	.	.	.	I	.	.	.	V	.	M	.	.	.	.	.	.	A	.	I	N	.	.	.
